# Effects of Shear Stress Waves on Meat Tenderness: Ultrasonoporation

**DOI:** 10.3390/foods12122390

**Published:** 2023-06-16

**Authors:** Raúl Alberto Reyes-Villagrana, Jesús Madrigal-Melchor, América Chávez-Martínez, Juliana Juárez-Moya, Ana Luis Rentería-Monterrubio

**Affiliations:** 1 IxM del CONAHCYT, Consejo Nacional de Humanidades, Ciencia y Tecnología, Ciudad de Mexico 03940, Mexico; 2 Unidad Académica de Ciencia y Tecnología de la Luz y la Materia, Universidad Autónoma de Zacatecas, Zacatecas 98000, Mexico; jmadrim@uaz.edu.mx; 3 Facultad de Zootecnia y Ecología, Universidad Autónoma de Chihuahua, Chihuahua 31453, Mexico; amchavez@uach.mx (A.C.-M.); jjmoya@uach.mx (J.J.-M.)

**Keywords:** acoustic cavitation, acoustic radiation force, *Longissimus dorsi*, meat, rib-eye, shear stress waves, ultrasonoporation

## Abstract

Meat is an important part of the food pyramid in Mexico, to such an extent that it is included in the basic food basket. In recent years, there has been great interest in the application of so-called emerging technologies, such as high-intensity ultrasound (HIU), to modify the characteristics of meat and meat products. The advantages of the HIU in meat such as pH, increased water-holding capacity, and antimicrobial activity are well documented and conclusive. However, in terms of meat tenderization, the results are confusing and contradictory, mainly when they focus on three HIU parameters: acoustic intensity, frequency, and application time. This study explores via a texturometer the effect of HIU-generated acoustic cavitation and ultrasonoporation in beef (m. *Longissimus dorsi*). Loin-steak was ultrasonicated with the following parameters: time t_HIU_ = 30 min/each side; frequency f_HIU_ = 37 kHz; acoustic intensity I_HIU_ = ~6, 7, 16, 28, and 90 W/cm^2^. The results showed that acoustic cavitation has a chaotic effect on the loin-steak surface and thickness of the rib-eye due to Bjerknes force, generating shear stress waves, and acoustic radiation transmittance via the internal structure of the meat and the modification of the myofibrils, in addition to the collateral effect in which the collagen and pH generated ultrasonoporation. This means that HIU can be beneficial for the tenderization of meat.

## 1. Introduction

The World Health Organization (WHO) has declared the conditions for maintaining a healthy diet [1]. This includes the Eatwell Plate, which describes the basic foods people need to maintain a balanced and nutritious diet [2,3]. The Eatwell Plate includes meat, which has been fundamental since the *Homo erectus* times for the development and growth of the population [4]. Meat consumption meant a change in human face anatomy, jawline, brain, and speech development. From then on, meat has been part of the human diet.

The concept of meat includes not only beef but veal, mutton, pork venison, squab, chicken, carabeef, chevon, and turkey, as well as meat from marine creatures. There are standards and procedures to study meat quality in addition to the implementation of various tools to meet this quality. Commonly, the consumer determines the quality traits a meat product should fulfill. However, three parameters are fundamental to deciding the final choice: color, juiciness, and tenderness [5].

The meat industry has developed and implemented quality and welfare-friendly slaughter and processing facilities, and they have pointed out where the new trends in terms of infrastructure should focus [6,7,8,9].

Research within the meat industry is mainly aimed at quality [5], predominantly meat tenderness. A variety of practices and processes to tenderize meat include diet modification in live animals [10], stimulation via electromagnetic waves [11], electric current [12], and microwaves [13], among others. Apart from the previously mentioned, there are hybrid processes, such as the generation of hydrodynamic shock waves by controlled explosions or hydrodyne [14,15,16,17].

Nowadays, the food industry has focused on green technologies [18], as well as the so-called non-thermal emerging technologies, to stimulate food, minimize damage, and improve quality [19].

Keeping the latter in mind, one of the most studied technologies regarding the quality, processing, extraction, and preservation of food has been high-intensity ultrasound (HIU) [20,21,22,23,24,25,26,27,28,29,30,31,32,33], also known as power ultrasound [34,35]. The advantages of HIU might surpass other technics due to the acoustic cavitation phenomenon, which causes the generation, growth, and collapse of air and/or vapor bubbles [36].

In meat, HIU has been used particularly to reduce microbial concentration [37], decrease temperature for meat conservation purposes [38], analyze meat quality [39], and support curing [40]. One of the most important parameters to study is tenderness, for example, toughness reduction, meat structure analyses under varying conditions [41,42,43], such as preservation [44,45,46,47] and marinade [48,49,50,51,52,53].

Nowadays, the use of HIU has not been optimized completely, and it has not reached its potential because the theory of ultrasound and the correct application of the technique have not been fully understood. The lack of understanding and the variation of acoustic intensities, frequencies, application times, media propagation, temperature, and types of ultrasonication instruments (such as ultrasonic baths and sonotrodes) reflect the ambiguous results [54,55,56].

The objective of this study was to examine the physical effect that shear stress waves have via the meat structure after ultrasonication in terms of color, pH, and shear force. 

## 2. Background

Meat is deemed as the voluntary striated skeletal muscles of animals, such as bovines, sheep, goats, pigs, horses, and birds, among others. However, seafood products, such as fish and shellfish, can also be considered meat [57]. The major components of meat are water, protein, lipids, and minerals, and their concentration (Table 1) depends on several factors, for example, diet, species, breed, etc.

**Figure 1:**
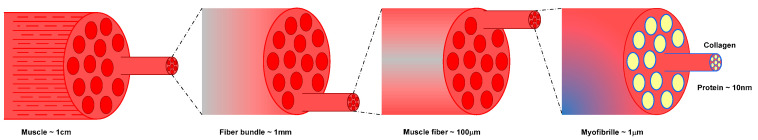
Composition of striated muscle.

The striated muscle has a fibrillar structure, and its physical characteristics depend on the physiology of the stress function. The smallest structures formed by the fiber bundles are the myofibrils (Figure 1) [59].

**Figure 2:**
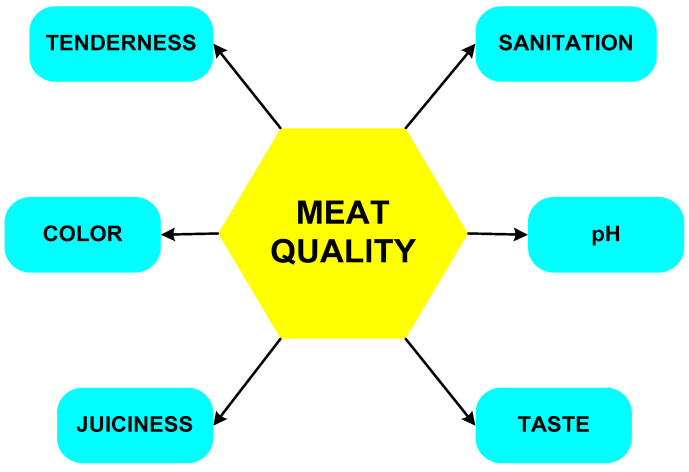
Meat quality traits.

The quality traits of meat are implicitly connected to the structure of the muscle and are based on six factors (Figure 2). Tenderness is one of the most important and studied. 

**Figure 3:**
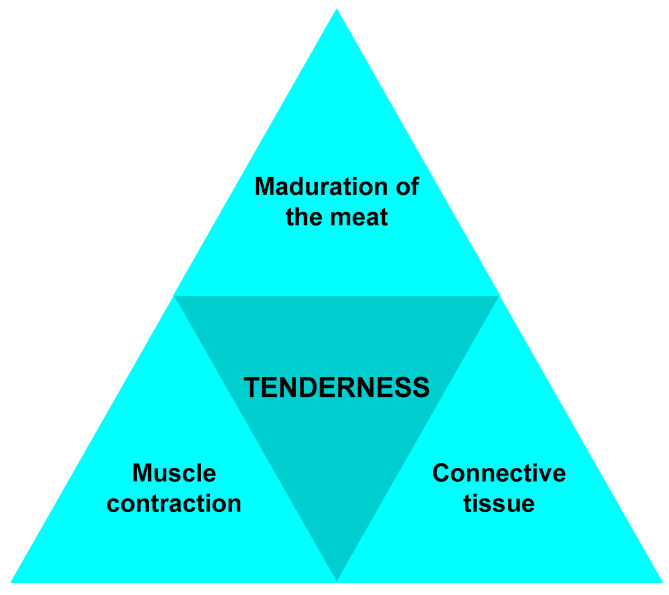
Factors influencing meat tenderness.

Tenderness is an important organoleptic criterion for consumers, and it has three main influencing factors: meat maturation, connective tissue, and muscle contraction (Figure 3) [60].

Tenderness may vary due to the amount of connective tissue and muscle myofibrils. Meanwhile, aging modifies the nutritional characteristics of meat and also benefits tenderness, reaching its maximum after 10 to 15 days of chilled storage (~0 °C). The normal maturation process can be altered if the muscle rapidly changes its thermodynamic conditions after slaughter, thus affecting muscle contraction, thereby increasing hardness.

The mechanical properties of meat (toughness/tenderness) can be assessed using several methods, such as cutting (Warner-Bratzler and Kramer), compression (Minrinz and Volodkevich), tension (Staples), penetration (Armour), grinding, and fragmentation [61].

There are different methodologies to decrease toughness [62] and increase tenderness (Table 2), including non-thermal technologies [18,63], such as HIU.

Studies in Table 2 are mostly based on beef, pork, chicken, and, in a few cases, seafood. The ultrasonicated meat is either unpacked or vacuum-packed (and marinated). The tenderness is mainly determined using the Warner-Bratzler shear force technique and complemented with electron microscopy, nuclear magnetic resonance, texture profile analyses, transmission electron microscopy, etc., and the data is analyzed primarily using statistical methods. The results (Table 2) are ambiguous, as tenderness is affected positively and negatively, or it was not affected at all.

Almost all studies indicate acoustic properties, such as intensity, frequency, and application times (including times applied to each side on meat fillets), although few of them indicate the temperature. Likewise, the equipment was barely reported. For example, the most recent studies describe the use of ultrasonic baths without including further details. 

HIU is a very important tool for the treatment and transformation of the physical, chemical, and biological properties of food. In [96,97], they extensively describe ultrasound and the effect of acoustic cavitation.

### Ultrasonoporation

**Figure 4:**
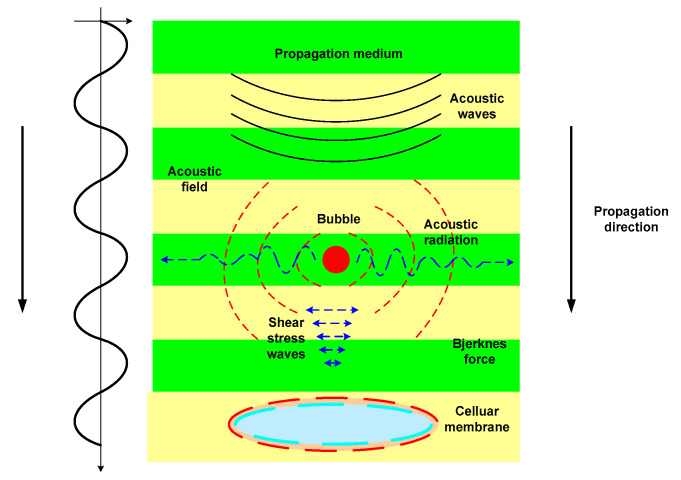
Depiction of ultrasonoporation.

Ultrasonoporation is interpreted as a methodology that involves the effect of acoustic cavitation generated via HIU to modify the internal structure of biological materials, both animal and plant, on a cellular scale. The most common applications are given in the introduction of drugs and tissue regeneration [98]. Ultrasonoporation alters the permeability of the cell plasma membrane via shear stress waves. The Bjerknes force, which is divided by the primary force generated via the acoustic radiation of the bubble in an acoustic field and the secondary force caused by the interaction between two bubbles [99,100], is induced by acoustic cavitation, as seen in Figure 4.

## 3. Materials and Methods

The effects of shear stress waves via the internal structure of meat after ultrasonication were assessed on beef (m. *Longissimus dorsi*) 24 h *post mortem*. The beef came from a 24-month-old Angus × Hereford steer, grazed on native pastures until 18 months. Then, its diet was complemented for 2 months and finished under intensive conditions for 4 months. Then, the animal was slaughtered under national regulations in an abattoir. The carcass was transported to the University Meat Complex 24 h *post mortem*. m. *Longissimus dorsi* was divided into two parts: cranial and caudal. Then, 25.48 ± 1.01 mm-tick steaks with the fibers perpendicular to the cut were obtained from each part. The meat samples were measured with a vernier (Mitutoyo^®^). Finally, the steaks were vacuum packed and stored for 24 h at 4 °C. Subsequently, the fillets were removed from their packaging and left at room temperature (~18 °C and at an atmospheric pressure of 1018.3 hPa) until equilibrium was reached. 

### 3.1. Ultrasonic Treatment

**Figure 5:**
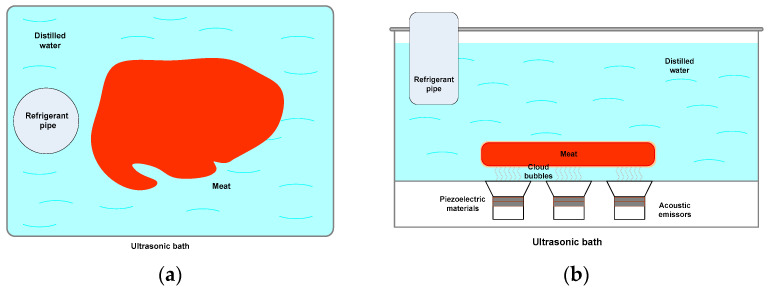
Experimental setup: (**a**) top view and (**b**) lateral view.

Ultrasonication treatments were randomly assigned to each sample. Samples were obtained from the central part of the steak (rib-eye). Prior to the ultrasonic treatments, fat and connective tissue were removed. Treatments were elaborated by applying acoustic intensities of 6, 7, 16, 28, and 90 W/cm^2^ with a frequency of 37 kHz, using ultrasonic baths (ELMASONIC^®^, Singen, Germany). One steak was used as a control sample (without ultrasonication). The ultrasonication time was 60 min (30 min/for each side of the sample), and the temperature of the ultrasonic baths was controlled via a refrigerant system (Julabo^®^, model: FT200, Seelbach, Germany) at 4 °C. The samples (rib-eye) were placed one by one in 0.5 L of distilled water in each ultrasonic bath, as seen in Figure 5.

### 3.2. Color Measurement

Meat color was determined instrumentally with a colorimeter (Konica Minolta^®^ Camera, UK, Aperture, 8 mm, Illuminant C, D_65_) before and after ultrasonic treatments. L, a*, b*, and C* were recorded directly using the colorimeter [101]. Measurements were made in triplicate in each sample. Sampling points were at the vertices of a triangular geometric shape.

### 3.3. pH Measurement

pH was determined instrumentally with a digital pH meter (Hanna Instrument^®^, HI99163, Nușfalău, Romania) before and after ultrasonic treatment. The instrument element was placed to a depth of 1.5 cm in the flesh at position of the vertices of a triangular geometric figure.

### 3.4. Shear Force Measurement

The steaks were cooked simultaneously on both sides, using an electric grill (George Foreman^®^, GR2080R, China), until reaching an internal temperature of 71 °C in the center. Later these were cooled on an ice bed. Ten 8 mm diameter and 10 cm long cylinders were obtained from the rib-eye of each treatment. The cutting force was analyzed with a TA-Tx plus texturometer (Stable Micro Systems^®^ Ltd., Surrey, UK) and cut with a Warner-Bratzler blade, with a speed of 2 mm/s. Peak force and positive areas were recorded in the system. The results were analyzed in the OriginLab^®^ 8.0 software from OriginLab Corp. 

### 3.5. Statistical Analysis

The design was proposed as a factorial randomized block system 2 × 5 (2 parts of the loin and 5 acoustic intensities) of ultrasonication treatment. The system involves the part of the loin and the acoustic intensities that induce the modification of meat quality variables (color, pH, and shear force). It was analyzed using a generalized linear model. Tukey’s tests were applied to evaluate significant differences among means (α = 0.05). The software SAS-STAT^®^ 9, 2002 was used. 

## 4. Results and Discussion

### 4.1. Color

**Figure 6:**
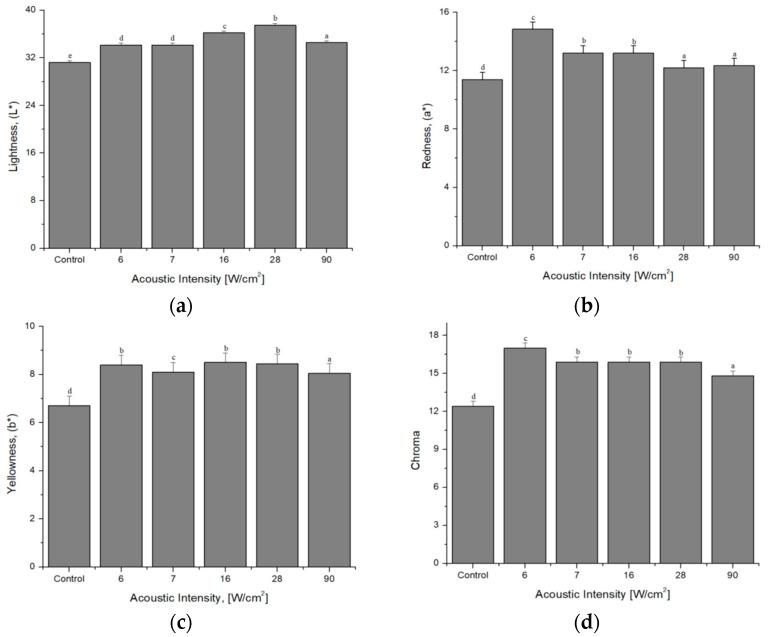
Color analysis: (**a**) lightness (L*), (**b**) redness (a*), (**c**) yellowness (b*), and (**d**) chroma. ^a,b,c,d^ Different letters indicate significant differences (*p* < 0.05).

In terms of meat, lightness (L*), is the light-reflecting capacity of the tissue surface. The results showed a statistical difference in lightness (*p* < 0.0001) among treatments. The control sample presented the lowest L* values. Intermediate acoustic intensities (16 and 28 W/cm^2^) increased lightness (see Figure 6a). It is considered that there was a superficial rupture of the structure of the samples caused by the shear stress waves. Because of the transient cavitation in cloud and filament mode colliding on the surface of the samples, where the Bjerknes force is implicated, this causes loss of water on the surface of the samples and increased lightness. Some authors [37] report similar results where the ultrasonication time is similar or longer. However, it is not clear how they controlled the parameters that influence the environment during the treatment process. Likewise, the authors do not describe how they control the increase in ultrasonication temperature. It should be noted that the increase in lightness cannot be considered beneficial for meat quality. 

With regard to redness (a*), treatments were statistically different (*p* < 0.0001). Redness is the most important parameter for meat a*, which intrinsically implies an undesirable color and rejection by consumers [102]. Redness is directly correlated with myoglobin; low acoustic intensities (6 and 7 W/cm^2^) increased a* values (see Figure 6b). The acoustic intensity with 16 W/cm^2^ is proportional to increasing the L* and the redness of the samples.

The yellowness (b*) of the meat samples showed a statistical difference (*p* < 0.0001). The acoustic intensities applied within the lower and medium range via the ultrasonic baths (6, 7, and 28 W/cm^2^) influenced and increased the b* in the samples (see Figure 6c). This could be, associated with ultrasonoporation, as it directly affects the redox state of myoglobin in samples [103]. The increased b* of the meat has been related to brown colorations [104]. However, higher b* values can be ambiguous, so it can be a disadvantage since the brown colorations of the meat can cause consumer rejection [105].

The saturation or chroma index represents the color intensity of an object and includes the coordinates a* and b*, where it represents the magnitude of the variable (see Figure 6d). For this variable, a statistical difference was observed among treatments (*p* < 0.0001). For the low acoustic intensities generated via the ultrasonic baths (6 and 7 W/cm^2^), an increase was shown towards the a*; this is due to the fact that the shear stress waves and the second strength of Bjerknes are mild intensity in the surface erosion in meat samples. It has been mentioned that chroma values below 18 are more likely to be rejected by consumers [106]. It is well known that the red and chroma variables are the most relevant to achieve the cherry red color sought by consumers in the market [107].

### 4.2. pH Measurement

**Figure 7:**
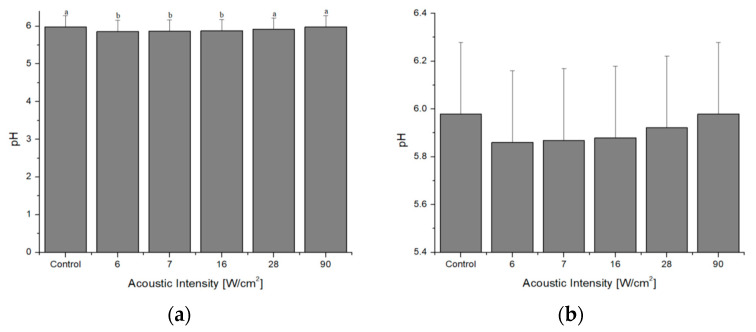
(**a**) pH analysis and (**b**) pH detail. ^a,b^ Different letters indicate significant differences (*p* < 0.05).

Ultrasonoporation causes a decrease in pH (*p* = 0.0134) (see Figure 7) and homogenizes its values, regardless of the acoustic intensity. The shear stress waves induce the breaking of the hydrogen bonds of the water molecules and generate an increase in radicals. However, the results are uncertain as some conclude that HIU did not influence pH [71] due to the cell structure damage that releases ions and hides acid groups [84]. Regarding the myofibrillar proteins, the higher pH has been attributed to the denaturation of the proteins and to the free production of radicals, which interact with the protein side chains [46].

### 4.3. Shear Force Measurement

**Figure 8:**
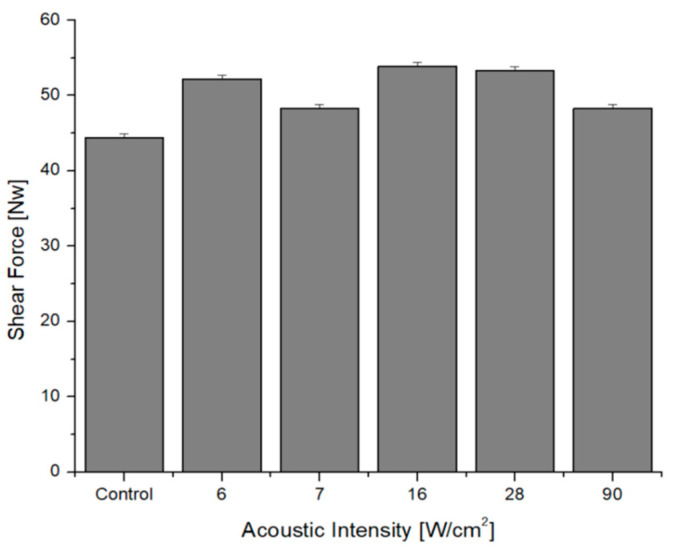
Shear force analysis.

The shear force did not present statistical differences (*p* = 0.0544), including those of the cranial and caudal parts (see Figure 8). Shear stress waves influence the erosion of the meat surface. It may be of interest if the working frequency of the ultrasonic baths were modulated. In other words, as the frequency rises, the number of bubbles increases, but their size decreases. Consequently, the first and second strengths of Bjerknes would present a benefit in the tenderness of the meat. However, it has not been shown how high-intensity acoustic transmittance can exist via the meat structure. Even so, there is controversy because some studies do not report a softness effect with HIU [43,71,76], while others have found effects on tenderness, depending on whether cooking was within the range between 50 °C and 70 °C [24,43,56].

Upon reviewing the results described in Table 2, as well as the results obtained in this present study, it is necessary to delve into the following points:

1. The effects of ultrasound on meat tenderness are mainly examined on bovines, poultry, and, in the least cases, seafood. Muscles in beef have different internal anatomical structures. *Longissimus dorsi*, for instance, has almost all its myofibrils arranged collinearly, thus facilitating the internal exploration of its structure. Other muscles are organized transversally or randomly. The organization of the muscles impacts the results of the Warner-Bratzler technique and the sensory analyses. There is a variation in the mechanical stress–strain relationship of the muscles and a great influence in those that have a greater concentration of connective tissues and bone.
2. The ultrasonic bath and the sonotrode-type ultrasonicator are the most used apparatus. The ultrasonic bath has up to three acoustic emitters attached to the bottom of the ultrasonic basket, and, due to its design, it has a baffle-type acoustic radiation behavior and, consequently, randomly generates clouds of filament-type microbubbles over the entire surface of the ultrasonication basket. The sonotrode-type ultrasonicator is placed directly in the fluid to be ultrasonicated. The emission generated by the ultrasonicator, depending on the acoustic intensity and the type of sonotrode, is an inverted pyramidal or half-sphere microbubble cloud, which subsequently leads to the generation of filament-shaped microbubble clouds. In both ultrasonication systems, the acoustic intensities and the frequency of the ultrasonicator vary.
3. HIU has the greatest application impact on the surface of the material. When applying this technique on meat foods but theoretically ignoring the basics of ultrasonication, different hypotheses or questions will be raised, such as those concerning the application of HIU on both sides of a meat cut or/and increasing the application times. As described in Table 2, most studies similarly vary on the types of muscle, packing (packed or unpacked), or processing (marinated foods). These modifications usually are conducted unaware of the acoustic and thermal properties of the system and the environment, which notably influence the experimental results.
4. Meat products can be studied as deformable materials, and their behavior might be analyzed with time-dependent models, such as Newton, Maxwell, Kelvin, Burger, and Bingham, or their combinations, stimulated via an acoustic source. Then, these results can be correlated with the results from the Warner-Bratzler technique.
5. Previous knowledge of acoustic, mechanical, and thermal properties of the ultrasonication system, as well as of the meat products, would facilitate the prediction of the results and their discussion. The characteristics of acoustic physics in HIU are more than the acoustic intensity and frequency, which describe the speed that the ultrasound pressure exerts, the number of microbubbles generated, as well as the size of them. The experimental methodology should include the control of the variables that can affect this study, for example, the space where the experiments will be carried out, since it may compromise the temperature and atmospheric pressure. Additionally, the volumetric density properties of the fluid should be determined. The vibration generated from the acoustic emitters on the basis (bottom) of the ultrasonic bath and the implosions of acoustic cavitation in a stable and transitory state cause increases in temperature via convection. This influences the variation of thermal wave diffusion that finally reaches a well-defined temperature gradient point. Other studies circulated the fluid to cool it down. However, these settings make the acoustic cavitation shift to hydrodynamic cavitation and the conditions of the experiment change. In this sense, it is important to determine the acoustic field of application, either Fresnel or Fraunhofer. In the studies described in Table 2, the position of the meat with respect to the acoustic emitter is not mentioned. This detail is relevant as the effect of the acoustic radiation on the meat can be defined.
6. Studies on vacuum-packed meat and meat products did not favor tenderness. This might be obvious, as there is no effect of acoustic cavitation via vacuum packaging. In acoustic physics, meat wrapped with a polymer resembles several interphase systems. The acoustic wave will travel through it; thus, the acoustic transmission effect is presented [108]. HIU applied to vacuum-packed meat has no effect on the internal structure of the meat, as the acoustic radiation generated by the shock waves does not pass through the interphase of the package; these are reflected. However, if the meat is packed with brine (or marinated), the acoustic waves have a propagation medium. So, in this case, the acoustic cavitation will influence the structure of the meat. However, the meat and the marinade should not be vacuum-packed, as there will be no effect of the ultrasound on the product.

## 5. Conclusions

HIU is an emerging tool applied to the treatment and preservation of food products that, given the phenomenology that causes acoustic cavitation, has special effects that provide benefits in food quality, such as ultrasonoporation and shear stress waves. In this study, an analysis of HIU applied to meat products was presented. The effects on bovine meat, specifically the *Longissimus dorsi* muscle, were studied. The color, pH, and tenderness properties of the steaks were analyzed. Various ultrasonics baths were used, with a variety of acoustic intensities and a single working frequency, and a 60 min treatment (30 min/side of each sample). The results indicate that the treatment with low acoustic intensities of 6 and 7 W/cm^2^ had a favorable effect on the meat color parameter, increasing L*, the saturation index, and a*. Meanwhile, the pH remained quasi-stable at all applied acoustic intensities. There were no favorable results in tenderness. It should be recommended that if intended to continue exploring the effect of HIU on meat products, several variables must be controlled, such as the amplitude and frequency of the acoustic wave. 

## Figures and Tables

**Table 1:** Average composition of various types of meat [58].

Component (%)	Meat Type						
	Beef	Pork	Mutton	Chicken	Turkey	Tuna	Mojarra
Water	71	68	60	66	70	69	79
Protein	21	13	19	18	21	24	17
Lipids	7	18	20	15	8	6	3
Minerals	1	1	1	1	1	1	1

**Table 2:** Effects of high-intensity ultrasound in meat products.

Sample Description	Ultrasonic Parameters (Intensity, Frequency, Time, Temperature)	Texture—Tenderness ^1^	Analyses to Demonstrate Changes ^2^	Observations	Ref.
Soluble beef collagen	50 W, 9 kHz, 10 to 440 min; 8–11 °C.	-	SA ACP	Molecular weights and hydrodynamic conditions were reviewed Viscosity reduction	[64]
Pork Cured ham Cylindric samples 25 long × 3.5 cm diameter	40 kHz; 15, 30, 60, and 120 min.	Instron universal testing machine	SA MI	Effects of muscle microstructure, breaking force, cooking performance, and protein stability	[65]
Beef m. *Semitendinosus* 10 × 5 × 2.5 cm sample size	1000 W, 25.9 kHz, 0, 2, 4, and 8 min (longitudinal cuts); 0, 2, 4 8 and 16 min (cross sections).	WB	SA	Increase in tenderness in 2 and 4 min	[66]
Beef m. *Biceps pectoralis*	22 W/cm^2^; 20 kHz; 0, 5, and 10 min	WB	SA	Low effect on tenderness	[67]
Beef Vacuum packed m. *Semitendinosus* 2.5 × 6.4 × 10.2 cm 2.5 × 5.1 × 10.2 cm m. *Biceps femoris* 1.3 × 7.6 × 10.2 cm	1.55 W/cm^2^; 20 KHz; 8, 16, and 24 min. 1.55 W/cm^2^; 2 kHz; 30 min (15 min/side).	WB	SA	No effect on tenderness	[68]
Beef Vacuum packed m. *L. thoracis lumborum*, *Semimembranosus*, *Biceps femoris*	Hilsonic: 0.29 W/cm^2^; 47 kHz. Kerry: 0.39 W/cm^2^; 34–42 kHz. Ultrawave: 0.62 W/cm^2^; 30–40 kHz.	Bite force trendometer	SA	No major effects on tenderness	[69]
Beef m. *Semimembranosus*	10 W/cm^2^; 2.5 MHz; 2, 6, and 15 s.	Universal testing machine	SA MI	Unfavorable effects on tenderness	[70]
Beef m. S*emitendinosus* and *Longissimus* 60 mm × 40 cm × 20 mm	12 W/cm^2^; 24 kHz; 4 min.	WB	SA	Beneficial effects on tenderness, including maturity time of 3 and 7 days	[71]
Beef m. *Semimembranosus* 70 × 70 × 80 mm	2 W/cm^2^; 4 kHz; 2 min.	Nuclear magnetic resonance	SA MI	Favorable and unfavorable results	[72]
Beef m. *L. lumborum* pre- and post-*rigor*	48 kPa–65 kPa at 600 kHz; 48 kPa at MHz.	Tenderometer	SA	Unfavorable effects	[73]
Chicken Breast 2.5 × 5.5 × 1.0 cm	1500 W; 40 kHz, 30 or 60 min	Transmission electron microscopy	ACP SA MI	Favorable effects	[74]
Beef Vacuum packed m. S*emitendinosus* 2.5 × 5.0 × 5.0 cm	1500 W; 40 kHz; 10, 20, 30, 40, 50, or 60 min; 20 °C.	WB Optical microscopy SEM	SA MI	Favorable effects	[75]
Molluscs 2 × 2 × 2 cm	100–250 W; 45 kHz; 2–16 min; 10–60 °C.	WB SEM	SA MI Sensory evaluative	Favorable effects	[76]
Beef Lean (100 g)	1000 W; 25 kHz; 60% amplitude 5.5 min, 10 °C.	TPA	SA ACP	Favorable effects	[77]
Beef Lean (100 g)	230 W; 25 kHz; 60% amplitude 0, 9, and 18 min.	TPA	SA Sensory evaluative	Unfavorable effects	[78]
Chicken Breast 4 × 4 × 2 cm	350 W; 20 kHz; 5 min.	TPA SEM	SA MI	Favorable effects	[79]
Beef m. *L. lumborum* 13 × 9 × 2.5 cm *Infraspinatus, Cleidooccipitalis* 6 × 7 × 2.5	11 W/cm^2^; 40 kHz; 0, 40, 60, and 80 min.	WB SEM	SA MI	Favorable effects for some muscles and not for others	[80]
Beef m. *L. dorsi* 20 × 50 × 10 mm	150 and 300 W, 20 kHz; 30 and 120 min.	WB Electronic microscopy via transmission	SA MI	Increase tenderness	[81]
Beef m. *L. lumborum* 3 × 3 × 3 cm	100 and 300 W, 20 kHz; 10, 20, and 30 min, 11–17 °C.	WB Optical microscopy	SA MI	Favorable effects	[82]
Beef m. *L. dorsi*	11 W/cm^2^, 40 kHz; 60 min.	WB	SA Sensory evaluative	Benefits tenderness	[83]
Beef Vacuum packed m. *Semitendinosus* 80 × 70 × 25 mm	25 W/cm^2^, 20 kHz; 20 or 40 min.	WB Transmission electron microscopy	SA IEM	Benefits tenderness	[84]
Beef m. *L. lumborum* 3 × 3 × 3 cm	100 and 300 W, 20 kHz; 10, 20, and 30 min. Pulse train	Light microscopy SEM	SA IEM	Benefits tenderness combined with papain	[85]
Beef Flanks 8 × 8 × 8 cm	0, 400, 600, 800, and 1000 W, 20 kHz; 80, 100, and 120 min.	TPA Nuclear magnetic resonance Transmission electron microscopy	SA IEM	Benefits tenderness with powers > 800 W and 120 min	[86]
Chicken Breast	300 W, 40 kHz; 0, 10, 20, 30, and 40 min.	TPA SEM Nuclear magnetic resonance	SA IEM	There were no beneficial effects	[87]
Pork m. *Semitendinosus* 60 × 100 × 20 mm	90 and 54.9 W/cm^2^, 20 kHz; 120 min.	WB Nuclear magnetic resonance	SA	Benefits tenderness, with intensity 54.9 W/cm^2^	[88]
Fish fillets *Pangasius hypothalamus* and *Oreochromis niloticus* 10 × 10 × 10 mm	150 W, 40 kHz; 15 min.	TPA SEM	SA IEM	Unfavorable effects	[89]
Seafood Silver carp Surimi gel	300 W; 25, 45, 80, and 130 kHz.	Acoustic intensity measurements	SA	As the acoustic intensity increases, the force increases	[90]
Rabbit	110 W; 40 kHz; 0 and 120 min; 4 °C.	TPA	SA	Increased toughness	[91]
Beef Vacuum packed m. *L. lumborum, Semitendinosus* 10 × 89 × 2.5 cm	16 W/cm^2^, 28 W/cm^2^; 37 kHz; 40 min (20 min/side), 5 °C.	WB	SA	Unfavorable effects	[92]
Beef m. *L. lumborum* 10 × 5 × 2.5 cm	90 W/cm^2^; 37 kHz; 0, 10, 20, or 40 min; 4 °C.	WB	SA	Tenderness benefits in times of 40 min	[93]
Rabbit Vacuum packed m. *L. dorsi, Semimembranosus, Semitendinosus*	12 W/cm^2^; 24 kHz; 15 min; 5 °C.	WB	SA	Favorable effects on tenderness	[94]
Beef Vacuum packed m. *L. lumborum* 2.5 cm thick	90 W/cm^2^; 37 kHz, 40 min/side, 4 °C.	WB	SA	Unfavorable effects on tenderness	[95]

^1^ WB = Warner–Bratzler shear force, SEM = Scanning Electron Microscopy, TPA = Texture Profile Analysis. ^2^ SA = Statistical Analysis, ACP = Analytical Chemical Procedures, MI = Microscopy Image, IEM = Images via Electron Microscopy.

## Data Availability

The data used to support the findings of this study can be made available by the corresponding author upon request.
